# Sensitivity of various adiposity indices in identifying cardiometabolic diseases in Arab adults

**DOI:** 10.1186/s12933-015-0265-5

**Published:** 2015-08-07

**Authors:** Nasser M Al-Daghri, Omar S Al-Attas, Kaiser Wani, Abdullah M Alnaami, Shaun Sabico, Abdulrahman Al-Ajlan, George P Chrousos, Majed S Alokail

**Affiliations:** Biomarkers Research Program, Biochemistry Department, College of Science, King Saud University, Riyadh, 11451 Kingdom of Saudi Arabia; Prince Mutaib Bin Abdullah Chair on Osteoporosis, Biochemistry Department, College of Science, King Saud University, PO Box, 2455, Riyadh, 11451 Kingdom of Saudi Arabia; Department of Clinical Lab Sciences, College of Applied Medical Sciences, King Saud University, Riyadh, 11451 Kingdom of Saudi Arabia; First Department of Pediatrics, Athens University Medical School, 11527 Athens, Greece

**Keywords:** Visceral adiposity index, Body adiposity index, DMT2, Cardiometabolic diseases, Arabs

## Abstract

**Background:**

Obesity is a recognized risk factor for various cardiometabolic diseases and several indices are used clinically to assess overall cardiometabolic risk. This study aims to determine the sensitivity of six anthropometric indices [Body mass index (BMI), waist, waist-to-hip ratio (WHR), waist-to-height ratio (WHtR), body adiposity index (BAI) and visceral adiposity index (VAI)] in determining diabetes mellitus type 2, coronary heart disease, dyslipidemia, hypertension and metabolic syndrome (MetS) in Saudi adults recruited from two independent cohorts (2008–2009 and 2013–2014).

**Methods:**

A total of 6,821 Saudi adults [2008–2009, N = 3,971 (1,698 males and 2,273 females); 2013–2014, N = 2,850 (926 males and 1,924 females)] aged 18–70 years old were included in this descriptive, cross-sectional study. Anthropometrics were obtained and fasting blood samples analyzed for glucose and lipids. BMI, WHR, WHtR, BAI and VAI were computed mathematically.

**Results:**

VAI was the most sensitive index in determining DMT2 (AUC 0.72; p < 0.001) in the 2008–2009 cohort and MetS (AUC = 0.84; p < 0.001) in the 2013–2014 cohort. WHR was most discriminating for CHD in both cohorts (AUC 0.70 and 0.84 for 2008–2009 and 2013–2014, p values <0.001, respectively). WHtR was most sensitive but rather modest in determining hypertension (AUC 0.66; p < 0.001), while waist circumference was most sensitive for dyslipidemia (AUC 0.72; p < 0.001) in the 2008–2009 cohort and MetS (AUC 0.85; p < 0.001) in the 2013–2014 cohort. BAI was the least sensitive adiposity index.

**Conclusion:**

Sensitivity of adiposity indices regarding cardiometabolic diseases highlight the importance of body fat distribution in determining overall cardiometabolic risk, with indices involving abdominal obesity being more clinically significant than BMI and BAI. The sensitivity of these adiposity indices should be noted in assessing a particular cardiometabolic disease.

**Electronic supplementary material:**

The online version of this article (doi:10.1186/s12933-015-0265-5) contains supplementary material, which is available to authorized users.

## Background

Obesity is a major cardiovascular risk factor that has been a consistent global concern in modern time [1]. Recent epidemiologic evidence points to increased prevalence of overweight and obesity in most nations, but more notably in developing countries, with no reports of improvement since 1980 [2]. The Middle-Eastern region, Kingdom of Saudi Arabia (KSA) in particular, is not spared from this epidemic. It has been projected that obesity in KSA amongst adults will increase from 12% in 1992 to 41% by 2022 in males, and from 21 to 78% in females [3]. This observation is supported by a parallel increase in cardiometabolic diseases in the country [4].

Obesity has been conventionally defined as having a body mass index (BMI) of >30 kg/m^2^. Several anthropometric indices have also been used to define obesity based on fat distribution. These include waist circumference, waist-hip ratio and waist-to-height ratio, with each measure having their own advantages in predicting serious chronic non-communicable diseases and overall mortality [5–7]. Aside from these routine anthropometric indices, novel adiposity measures have also been proposed and are currently being tested in different populations. These include body adiposity index (BAI) proposed by Bergman and colleagues [8] and visceral adiposity index proposed by Amato and colleagues [9].

To date, BAI has been assessed in several populations as an alternative risk factor for several cardiometabolic diseases [10–12], while preliminary findings employing VAI as a surrogate marker for adipose tissue function hold promise mostly in insulin-resistance related diseases, such as diabetes mellitus type 2 (DMT2) [13, 14]. The comparison of BMI to the other adiposity indices mentioned above have not been carried out in the Arab population, while such studies have been done only partially in other ethnic populations. In fact, the combination of 5 adiposity indices (waist, WHR, WhTr, BAI and VAI) as opposed to BMI in determining hard outcomes have never been investigated in a large-scale setting. The present study, therefore, aims to identify the sensitivity of all five adiposity indices in determining DMT2, coronary heart disease (CHD), dyslipidemia, hypertension and metabolic syndrome (MetS) in the Arab population using two different cohorts gathered in 2008–2009 and 2013–2014.

## Methods

This is a descriptive, cross-sectional study involving two independent cohorts (2008–2009 and 2013–2014) of adult Saudis, which were obtained from the master database of the Biomarkers Research Program (BRP), College of Science, King Saud University (KSU), Riyadh, Saudi Arabia. A combined total of 9,769 subjects aged 18–70 years (N = 5,356 from the 2008–2009 cohort and N = 4,413 from the 2013–2014 cohort) were included. Ethics approval for both cohorts was obtained from the Ethics Committee of the College of Science, KSU, Riyadh, KSA.

### Subjects from the 2008–2009 cohort

Participants from the 2008–2009 group were part of a joint collaborative project between BRP and the Ministry of Health (Riyadh Cohort Database) consisting of more than 17,000 Saudis aged 1–70 years, who were randomly recruited from different households in the capital Riyadh, Saudi Arabia [4]. Non-ambulatory patients, expectant mothers and those that required immediate medical attention were excluded. A general questionnaire was administered to all participants with assistance from the research team. This questionnaire contained demographic information that included age and family history. Anthropometrics included height in meters and weight in kilograms, measured while patients were standing upright and with light clothing. Waist circumference was obtained using a standardized measuring tape rounded off to the nearest centimeter, measured midway between the lowest rib and iliac crest after normal expiration, while hip circumference was measured at the level of the greater trochanters as done previously [15]. Fasting blood samples were collected on the same day in the nearest primary care center. Written informed consent was obtained prior to inclusion.

### Subjects from the 2013–2014 xohort

Participants from the 2013–2014 group were taken from the on-going joint collaborative project between BRP and the Ministry of Education, which involved teachers and students from different public schools within Riyadh, Saudi Arabia, which began in 2013. A general questionnaire similar to that employed in the 2008–2009 cohort was administered and the same anthropometrics (height, weight, blood pressure, waist and hip circumference) were obtained following the same protocol of the 2008–2009 cohort. Fasting blood samples were taken by the school nurse. Non-consenting subjects were not included and similar exclusion criteria as in the 2008–2009 cohort were applied for participation in the study.

### Blood sample analyses

Fasting blood samples of both cohorts were analyzed and stored in BRP, KSU. In brief, all blood and serum samples were placed in plain polystyrene tubes, delivered on the same day at BRP and stored at −20°C. Fasting blood glucose and lipids (total cholesterol, triglycerides and HDL-cholesterol) were measured using a standard chemical analyzer (hexokinase and colorimetric methods, respectively) (Konelab, Vantaa, Finland) under strict conditions. The analyser was recalibrated frequently according to manufacturer’s instructions. LDL-cholesterol was estimated using the Friedwald equation [Total Cholesterol − (HDL − Cholesterol + (Triglycerides/2.2)] [16].

### Diagnosis of cardiometabolic diseases

Diagnosis of DMT2 was based on both the proposed cut-offs by the American Diabetes Association and by the World Health Organization (WHO) [fasting plasma glucose (FPG) level ≥7.0 mmol/L (126 mg/dL)] [17], as well as documented history of DMT2 (e.g., known DMT2 cases, use of anti-DMT2 drugs). CHD patients were known cases based on medical history of abnormal angiography, echocardiography, stress tests, history of cardiac catheterization or by-pass surgery and use of commonly used CHD medications (aspirin, β-blockers, ACE inhibitors, antiarrhythmic drugs, etc.). Adult hypertension was based on the consensus of the Seventh Joint National Committee on Prevention, Detection, Evaluation and Treatment of High Blood Pressure (≥140/90 mmHg) [18] and from use of anti-hypertensive medications. Dyslipidemia was diagnosed according to the National Cholesterol Education Program-Third Adults Treatment Panel (NCEP ATP III) [triglycerides ≥1.7 mmol/L with HDL cholesterol <1.03 mmol/L for men and <1.29 mmol/L for women]. Obesity was defined as BMI >30 kg/m^2^ [19]. Screening for MetS was done using the definition proposed by the International Diabetes Federation, which takes into consideration ethnic-specific cut-offs for waist circumference [20].

### Database cleaning and subject categorization

**Fig. 1:**
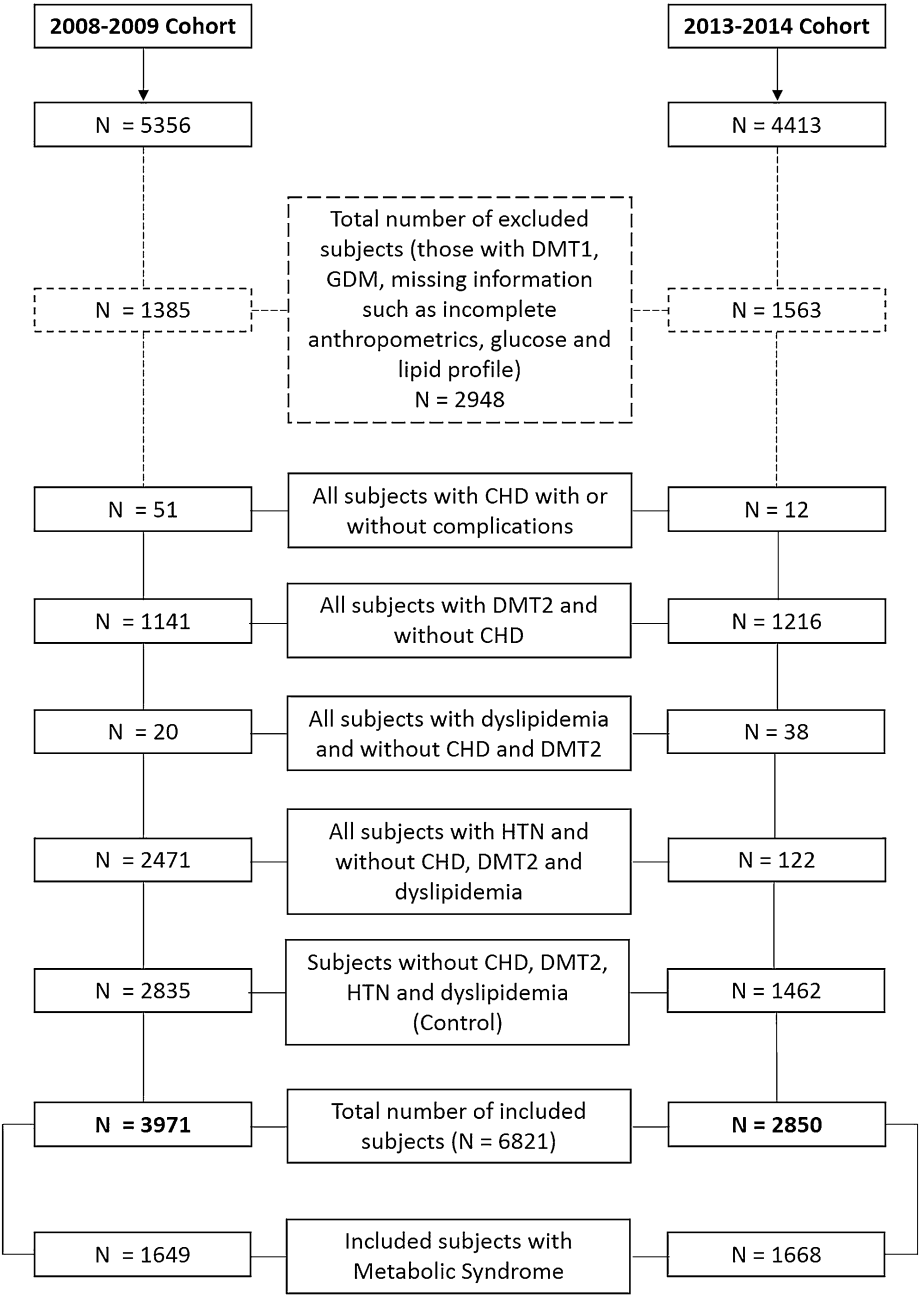
Flow diagram for the selection of subjects in both cohorts.

All subjects from both cohorts were initially categorized into 30 groups (Additional file 1: Table S1) based on the cardiometabolic disease present in each subject. These groups were downsized into five (Control, CHD, Diabetes, Dyslipidemia and HTN). Patients with DMT1 and GDM were excluded. All those with CHD as a single disease and with other comorbidities were combined as a single group. All those with DMT2 as a single disease and with other comorbidities except CHD were combined as a single group. All subjects with dyslipidemia and with other comorbidities except CHD and DMT2 were combined and all subjects who were hypertensive and/or obese were combined in one group. Obese or overweight subjects with none of the four cardiometabolic diseases were combined with controls. Subjects with missing information and with incomplete anthropometrics, glucose and lipid indices were excluded from the database, leading the final study sample size to N = 6,821 [2008–2009 N = 3,971 (1698 males and 2,273 females); 2013–2014 N = 2,850 (926 males and 1,924 females)]. Figure 1 shows the flow diagram of the breakdown of subjects.

### Adiposity indices calculations

Body mass index (BMI) was computed as weight (kg) divided by height in squared meters (m^2^). Waist-Hip ratio was obtained as the quotient of waist (cm) divided by hip (cm) circumferences. Waist to height ratio was calculated as waist (cm) over height (cm). Body adiposity index (BAI) was calculated as proposed by Bergman and colleagues [8] [hip circumference (cm) divided by (height (m)^1.5^ − 18]. Visceral adiposity index (VAI) was calculated based on the gender-dependent proposed formula by Amato and colleagues [9]:

$$\begin{aligned}{\text{VAI in Females}} &= \left[ {\text{Waist}}/\left\{ 3 6. 58 + \left( 1. 8 9\; \times \;{\text{BMI}} \right) \right\}\right]\\&\quad{\; \times \;} \left[{\text{Triglycerides}}/0. 8 1\right]\\&\quad {\; \times\;} \left[ 1. 5 2 / {\text{HDL}} - {\text{Cholesterol}} \right]\end{aligned}$$

$${\text{VAI in Males}} = \left[ {{\text{Waist/}}\{ 3 9. 6 8 + \left( { 1. 8 8\; \times \;{\text{BMI}}} \right)\} } \right] \, \times \, \left[ {{\text{Triglycerides}}/ 1.0 3} \right] \, \times \, \left[ { 1. 3 1/{\text{HDL}} - {\text{Cholesterol}}} \right]$$

### Data analyses

Statistical analyses were done using SPSS version 21.0 (SPSS Inc., Chicago, IL, USA). Continuous variables were presented as mean ± standard deviation and frequencies were expressed as percentages (%). All variables were checked for normality using the Kolmogorov–Smirnov test. Independent samples Student t test was done for comparisons between categorical parameters between cohorts. VAI was log transformed prior to t testing due to its non-Gaussian distribution. Bivariate Spearman’s correlation analysis was done to determine significant associations between cardiometabolic risk factors and adiposity indices while adjusting for multiple comparisons. Receiver Operating Characteristic (ROC) analyses were used to determine the area under ROC curves (AUC) between each cardiometabolic risk factor and adiposity index. P values for ROC were adjusted for multiple comparisons (Bonferroni corrected p value = 0.008). All tests were 2-tailed and p values were set depending on the threshold required to achieve a type 1 error rate of 0.05.

## Results

Table 1 shows the anthropometric and metabolic profile of the different cohorts. Prevalence of DMT2 and MetS were higher in the 2013–2014 than the 2008–2009 cohort (42.7 versus 28.7%, 58.5 versus 41.5%, respectively). Prevalence of other chronic diseases were low in both cohorts. The 2013–2014 cohort subjects were significantly older and had significantly higher mean adiposity indices, blood pressure, glucose, triglycerides and HDL-cholesterol than the 2008–2009 cohort (p values <0.001). The 2008–2009 cohort, on the other hand, had a significantly higher mean VAI and LDL-cholesterol than the 2013–2014 cohort (p values <0.001). The complete list of subjects based on their medical conditions is provided in Additional file 1: Table S1.

**Table 1:** General characteristics of subjects

Parameter	2008–2009	2013–2014	P value
N	3,971	2,850	
M/F	1698/2,273	926/1,924	
DM (%)	1,141 (28.7)	1,216 (42.7)	
CHD (%)	51 (1.3)	12 (0.4)	
Hypertension (%)	288 (7.3)	122 (4.3)	
Dyslipidemia (%)	20 (0.5)	38 (1.3)	
MetS (%)	1,649 (41.5)	1,668 (58.5)	
Age (years)	40.3 ± 16.2	47.3 ± 13.9	<0.001
BMI (kg/m^2^)	28.8 ± 6.3	30.6 ± 6.4	<0.001
Waist (cm)	89.6 ± 21.0	94.1 ± 21.8	<0.001
Hips (cm)	100.3 ± 21.5	102.3 ± 21.1	<0.001
WHR	0.91 ± 0.2	0.93 ± 0.2	<0.001
WHtR	0.55 ± 0.1	0.59 ± 0.1	<0.001
Body Adiposity Index	30.9 ± 13.5	34.3 ± 14.9	<0.001
Visceral Adiposity Index	3.8 ± 3.9	3.4 ± 2.9	<0.001
Systolic blood pressure (mmHg)	120.4 ± 15.2	126.2 ± 16.6	<0.001
Diastolic blood pressure (mmHg)	76.8 ± 9.0	80.0 ± 10.9	NS
Glucose (mmol/L)	6.9 ± 3.8	7.8 ± 4.0	<0.001
Triglycerides (mmol/L)	1.67 ± 1.0	1.84 ± 1.2	<0.001
Total cholesterol (mmol/L)	5.0 ± 1.2	5.0 ± 1.2	NS
HDL-cholesterol (mmol/L)	0.87 ± 0.4	1.0 ± 0.3	<0.001
LDL-cholesterol (mmol/L)	3.4 ± 1.0	3.1 ± 1.0	<0.001

Data presented as mean ± standard deviation; significant at p < 0.05.

Table 2 shows the bivariate associations of the different adiposity indices, including weight, to cardiometabolic risk factors in all subjects. Age was most strongly associated with WHtR (R = 0.47; p < 0.001) followed by waist circumference (R = 0.46; p < 0.001) and least with BAI (R = 0.16; p < 0.001). Height had the strongest inverse association with BAI (R −0.74; p < 0.001) followed by WHtR (R = −0.26; p < 0.001) and BMI (R = −0.19; p < 0.001). Both systolic and diastolic blood pressures were strongly correlated with waist circumference (R = 0.31 and 0.28, respectively) and weakly, with BAI (R = 0.06 and 0.05, respectively). Fasting glucose levels were significantly associated with all indices but strongest with waist circumference and VAI, both having a R = 0.34. As for the lipids, triglycerides, HDL-, LDL- and total cholesterol had the strongest associations with VAI (R = 0.77, −0.62, 0.16 and 0.21, respectively). Both triglycerides and total cholesterol had the weakest associations with BAI, while HDL-cholesterol had the weakest correlation with WHtR and BMI and LDL with WHR.

**Table 2:** Bivariate associations between adiposity indices and cardiometabolic risk factors

Parameter	BMI	Weight	Waist	WHR	WHtR	VAI	BAI
Age (years)	0.28**	0.19**	0.46**	0.44**	0.47**	0.31**	0.16**
Height (m)	−0.19**	0.33**	0.01	0.14**	−0.26**	0.06**	−0.74**
Systolic blood pressure (mmHg)	0.29**	0.27**	0.31**	0.29**	0.30**	0.19**	0.06**
Diastolic blood pressure (mmHg)	0.26**	0.27**	0.28**	0.23**	0.26**	0.17**	0.05**
Glucose (mmol/L)	0.24**	0.12**	0.34**	0.33**	0.33**	0.34**	0.10**
Triglycerides (mmol/L)	0.22**	0.19**	0.28**	0.28**	0.24**	0.77**	0.003
Total cholesterol (mmol/L)	0.16**	0.11**	0.14**	0.10**	0.15**	0.21**	0.08**
HDL-cholesterol (mmol/L)	−0.01	−0.12**	−0.06**	−0.14**	0.006	−0.62**	0.18**
LDL-cholesterol (mmol/L)	0.10**	0.08**	0.08**	0.03**	0.08**	0.16**	0.05**

Data presented as coefficient (R); ** denotes significance at 0.005 level.

The AUCs of the different adiposity indices of the two cohorts to the cardiometabolic diseases are presented in Table 3. The most sensitive adiposity index to determine DMT2 was VAI (AUC = 0.72) in the 2008–2009 cohort, while waist and WHR were the most sensitive anthropometric indices for DMT2 in the 2013–2014 cohort (AUCs = 0.70). WHR was consistently the most sensitive index to determine CHD in both the 2008–2009 and 2013–2014 cohorts (AUCs = 0.70 and 0.84, respectively). For hypertension, WHtR and BMI were more sensitive than other indices (AUCs = 0.66 and 0.58, respectively), but with poor predictability. Weight had the highest AUC for dyslipidemia (AUC = 0.73) in the 2008–2009 cohort, but none in the 2013–2014 cohort. Lastly, waist circumference had the highest AUC for MetS (AUC = 0.85) followed closely by WHtR and VAI for the 2008–2009 cohort, while in the 2013–2014 cohort VAI had the highest AUC (0.84), followed closely by waist circumference and WHtR, respectively.

**Table 3:** Receiving operator characteristics of various adiposity indices and cardiovascular diseases

	DM	CHD	HTN	Dyslipidemia	IDF (MetS)
2008–2009					
BMI	0.612 (0.59–0.63)**	0.585 (0.51–0.66)	0.658 (0.62–0.69)**	0.707 (0.61–0.81)**	0.736 (0.72–0.75)**
Weight	0.618 (0.60–0.64)**	0.56 (0.49–0.528)	0.652 (0.619–0.685)**	*0.730 (0.633–0.827)* **	0.74 (0.725–0.756)**
Waist	0.650 (0.63–0.67)**	0.664 (0.59–0.74)**	0.655 (0.62–0.69)**	0.715 (0.63–0.80)**	*0.851 (0.84–0.86)* **
WHR	0.678 (0.66–0.70)**	0.705 (0.64–0.78)**	0.607 (0.57–0.64)**	0.685 (0.60–0.75)**	0.688 (0.67–0.706)**
WHtR	0.646 (0.62–0.67)**	0.674 (0.60–75)**	0.663 (0.63–0.70)**	0.687 (0.61–0.76)**	0.848 (0.835–0.861)**
VAI	0.715 (0.70–0.73)**	0.599 (0.51–0.69)	0.562 (0.53–0.60)**	0.672 (0.59–0.75)**	0.814 (0.80–0.829)**
BAI	0.507 (0.48–0.53)	0.541 (0.45–0.63)	0.579 (0.54–0.62)**	0.499 (0.38–0.62)	0.659 (0.64–0.677)**
2013–2014					
BMI	0.596 (0.57–0.62)**	0.670 (0.55–0.79)**	0.583 (0.53–0.64)**	0.493 (0.41–0.57)	0.712 (0.69–0.733)**
Weight	0.585 (0.564–0.605)	0.351 (0.192–0.509)	0.569 (0.517–0.621)	0.465 (0.386–0.543)	0.675 (0.654–0.695)**
Waist	0.696 (0.68–0.72)**	0.795 (0.69–0.90)**	0.537 (0.48–0.60)	0.589 (0.52–0.66)	0.813 (0.795–0.832)**
WHR	0.695 (0.67–0.72)**	0.836 (0.72–0.95)**	0.494 (0.44–0.55)	0.589 (0.49–0.68)	0.70 (0.678–0.722)**
WHtR	0.688 (0.67–0.71)**	0.783 (0.69–0.88)**	0.553 (0.49–0.61)	0.586 (0.51–0.66)	0.808 (0.789–0.827)**
VAI	0.690 (0.67–0.71)**	0.800 (0.68–0.92)**	0.510 (0.45–0.57)	0.495 (0.42–0.57)	*0.837 (0.82–0.853)* **
BAI	0.563 (0.54–0.59)**	0.476 (0.37–0.58)	0.575 (0.52–0.63)	0.511 (0.42–0.61)	0.67 (0.644–0.688)**

Data presented as AUC (95% confidence interval); ** denotes significance at 0.008 level.

ROCs in italics denote most sensitive adiposity index for a given cardiometabolic disease.

## Discussion

We investigated for the first time the sensitivity of all five adiposity indices, including BMI, in assessing risk for DMT2, CHD, hypertension, dyslipidemia and metabolic syndrome in an adult Arab population on two separate large-scale cohorts. First we observed that VAI was most predictive of DMT2 and MetS as compared to the other indices, including BMI, in cohorts 2008–2009 and 2013–2014, respectively. Several studies on VAI confirm its significant association to glycemia-related biomarkers, including adipocytokines [13, 14] and has recently been proven to be a reliable indicator of overt metabolic syndrome [21]. While this finding is strongly in favor of VAI’s increasing clinical use, it is interesting to note that the concept of VAI for use in non-Caucasian populations needs to be further tested. VAI is a gender-specific model derived from BMI, waist circumference, triglycerides and HDL-cholesterol from healthy normal/overweight adult Caucasian populations [22], hence, its use in other ethnic groups, such as the ones used in this study should be interpreted with caution. The sensitivity of VAI to CHD was not far from WHR, especially in the 2013–2014 cohort and this was somehow expected, as several cases in the CHD group harbor DMT2. It has been reported that CHD is more severe in patients with DMT2 and VAI is strongly associated with the severity of CHD [23].

With regards to the best adiposity index for determining CHD, WHR was superior among other indices, confirming the recent report of Mousavi in a Middle-Eastern population that WHR change was associated with incident mortality, something not observed with BMI and waist circumference [24]. Our findings are also in accordance with several studies done using different populations, asserting that WHR is superior to other indices, such as BMI, in predicting mortality and cardiovascular events [25–27]. Furthermore, it is somehow expected that WHR and waist circumference, markers of abdominal obesity, together with other indices that utilized waist circumference as part of the mathematical model (WHtR and VAI) to be better in predicting harder outcomes related to vascular health than BMI and BAI [28], with waist circumference alone being sensitive in determining dyslipidemia and MetS.

As mentioned previously, several populations have assessed the clinical significance of the various anthropometric indices and attempted to determine which ones are best for use in the general population. In Singapore, where the general population was less heterogenous than that of our study, they found that BAI was not better than BMI or WHtR in identifying persons at risk for CVD [29]. This is in line with our present findings, where BAI was the least sensitive in determining the cardiometabolic diseases studied. Furthermore, we found that WHtR was most sensitive, albeit quite modest, in determining hypertension in the Arab population, at least in the 2008–2009 cohort. This finding also supports the notion that probably, at least in the Arab population, WHtR can also be used as a predictor for hypertension, as several large-scale studies done in different ethnic groups also attest its superiority over BMI (and waist circumference) for detecting cardiometabolic risk factors [30–32].

It is important to highlight that the sensitivity of the various adiposity indices were determined and compared from two independent cohorts, with differences not only in time period but also in the subject selection. With the exception of WHR, which maintained supremacy over other indices in predicting CHD, overall, there was no “best” index, as the sensitivity levels of the adiposity indices were modestly near one another. Furthermore, VAI as a visceral fat function indicator is the only index used which contains blood parameters that directly affect variance in insulin resistance [33], hence, the bias in comparison to anthropometric indices. What the present study clearly suggests is that measures of abdominal adiposity maybe more clinically relevant than BMI and BAI in their ability to detect cardiometabolic diseases in the Arab ethnic population. Abdominal fat accumulation has been consistently linked to cardiovascular prognosis and vascular complications [34, 35]. In particular, visceral adiposity influences vascular health in its association with incident hypertension and its close associations to the cardiometabolic complications of obesity [36, 37], making it a promising target of therapy for cardiometabolic diseases [38].

The authors acknowledge several caveats. The downsizing of the 30 original groups to 5 may have created bias in the sensitivity of adiposity indices. While it may have been ideal to assess the different diseases as a single entity without any comorbidity, the intention was to maximize the sample size since the other groups were smaller and combining them into a bigger group based on the presence of a single hard outcome (CHD, DM, etc.) may hold more strength than simply excluding the group from the analysis. Also, the findings did not include other relevant factors such as physical activity and diet, as well as other biomarkers of adiposity and diagnostic markers (HbA1c and imaging tests) in the assessment of adiposity indices. Nevertheless, the findings are robust and support other several large-scale studies that highlight the clinical significance of the various anthropometric parameters commonly used in epidemiologic studies. It is also the first of its kind in the Arab population, where the incidence of cardiometabolic diseases are more common than in other groups, reinforcing ethnic-specific differences in the manifestation of insulin resistance-related diseases [39].

In summary, we determined for the first time the sensitivity of 6 anthropometric indices (BMI, waist, WHR, WHtR, BAI and VAI) in determining cardiometabolic diseases in the adult Arab population. In at least one cohort, VAI is most sensitive in determining DMT2 and MetS (together with waist circumference), WHR in CHD, WHtR in hypertension and waist circumference in dyslipidemia, highlighting the importance of body fat distribution, abdominal obesity in particular, in assessing overall cardiometabolic risk. These screening tools should be promoted for clinical use and their predictive values noted in assessing at risk Arab adults.

## Additional file

Additional file 1:
**Table S1.** Subject classification distribution according to cohort.
