# Supplementary material for: Sensitivity of various adiposity indices in identifying cardiometabolic diseases in Arab adults
Source: Cardiovasc Diabetol. 2015 Aug 7;14:101. doi: 10.1186/s12933-015-0265-5 (PMC4566864; doi:10.1186/s12933-015-0265-5)
Supplement: Additional file 1: — Table S1. Subject classification distribution according to cohort. [file 12933_2015_265_MOESM1_ESM.docx]

**Supplemental Table 1.** – Subject Classification Distribution According to Cohort.

|  | **All** | **2008-2009** | **2013-2014** |
| --- | --- | --- | --- |
| N | 9769 | 5356 | 4413 |
| Control | 3466 | 2019 | 1447 |
| CHD | 22 | 14 | 8 |
| CHD + Diabetes | 10 | 10 | 0 |
| CHD + Diabetes + Dys | 1 | 1 | 0 |
| CHD + Diabetes + Dys + HPN | 8 | 8 | 0 |
| CHD + Diabetes + Dys + HPN + Obese | 6 | 6 | 0 |
| CHD + Diabetes + HPN | 14 | 14 | 0 |
| CHD + Diabetes + HPN + Obese | 21 | 21 | 0 |
| CHD + Dys | 1 | 1 | 0 |
| CHD + Dys + HPN | 2 | 2 | 0 |
| CHD + Dys + HPN + Obese | 2 | 2 | 0 |
| CHD + Dys + Obese | 1 | 1 | 0 |
| CHD + HPN | 7 | 7 | 0 |
| CHD + HPN + Obese | 3 | 3 | 0 |
| CHD + Obese | 6 | 2 | 4 |
| Diabetes | 1691 | 805 | 886 |
| Diabetes + Dyslipidemia | 121 | 22 | 99 |
| Diabetes + Dyslipidemia + HPN | 21 | 21 | 0 |
| Diabetes + Dyslipidemia + HPN + Obese | 14 | 14 | 0 |
| Diabetes + Dyslipidemia + Obese | 75 | 12 | 63 |
| Diabetes + HPN | 405 | 242 | 163 |
| Diabetes + HPN + Obese | 237 | 237 | 0 |
| Diabetes + Obese | 1259 | 542 | 717 |
| Dyslipidemia | 81 | 34 | 47 |
| Dyslipidemia + HPN | 12 | 11 | 1 |
| Dyslipidemia + HPN + Obese | 17 | 13 | 4 |
| Dyslipidemia + Obese | 39 | 18 | 21 |
| HPN | 325 | 245 | 80 |
| HPN + Obese | 286 | 213 | 73 |
| Obese | 1616 | 816 | 800 |

**Note**: Data presented in frequencies (N)
